# Developing Message Strategies to Engage Racial and Ethnic Minority Groups in Digital Oral Self-Care Interventions: Participatory Co-Design Approach

**DOI:** 10.2196/49179

**Published:** 2023-12-11

**Authors:** Stephanie M Carpenter, Zara M Greer, Rebecca Newman, Susan A Murphy, Vivek Shetty, Inbal Nahum-Shani

**Affiliations:** 1 College of Health Solutions Arizona State University Phoenix, AZ United States; 2 Department of Psychology University of Wisconsin-Madison Madison, WI United States; 3 Oral and Maxillofacial Surgery School of Dentistry University of California, Los Angeles Los Angeles, CA United States; 4 Department of Statistics Harvard University Cambridge, MA United States; 5 Department of Computer Science Harvard University Cambridge, MA United States; 6 Institute for Social Research University of Michigan Ann Arbor, MI United States

**Keywords:** engagement, oral health, mobile health intervention, mHealth intervention, formative, racial and ethnic minority group, digital health, mobile health, mHealth, message development, health equity, racial minority, ethnic minority, digital intervention, dental care, barrier, oral self-care, mobile phone

## Abstract

**Background:**

The prevention of oral health diseases is a key public health issue and a major challenge for racial and ethnic minority groups, who often face barriers in accessing dental care. Daily toothbrushing is an important self-care behavior necessary for sustaining good oral health, yet engagement in regular brushing remains a challenge. Identifying strategies to promote engagement in regular oral self-care behaviors among populations at risk of poor oral health is critical.

**Objective:**

The formative research described here focused on creating messages for a digital oral self-care intervention targeting a racially and ethnically diverse population. Theoretically grounded strategies (reciprocity, reciprocity-by-proxy, and curiosity) were used to promote engagement in 3 aspects: oral self-care behaviors, an oral care smartphone app, and digital messages. A web-based participatory co-design approach was used to develop messages that are resource efficient, appealing, and novel; this approach involved dental experts, individuals from the general population, and individuals from the target population—dental patients from predominantly low-income racial and ethnic minority groups. Given that many individuals from racially and ethnically diverse populations face anonymity and confidentiality concerns when participating in research, we used an approach to message development that aimed to mitigate these concerns.

**Methods:**

Messages were initially developed with feedback from dental experts and Amazon Mechanical Turk workers. Dental patients were then recruited for 2 facilitator-mediated group webinar sessions held over Zoom (Zoom Video Communications; session 1: n=13; session 2: n=7), in which they provided both quantitative ratings and qualitative feedback on the messages. Participants interacted with the facilitator through Zoom polls and a chat window that was anonymous to other participants. Participants did not directly interact with each other, and the facilitator mediated sessions by verbally asking for message feedback and sharing key suggestions with the group for additional feedback. This approach plausibly enhanced participant anonymity and confidentiality during the sessions.

**Results:**

Participants rated messages highly in terms of liking (overall rating: mean 2.63, SD 0.58; reciprocity: mean 2.65, SD 0.52; reciprocity-by-proxy: mean 2.58, SD 0.53; curiosity involving interactive oral health questions and answers: mean 2.45, SD 0.69; curiosity involving tailored brushing feedback: mean 2.77, SD 0.48) on a scale ranging from 1 (do not like it) to 3 (like it). Qualitative feedback indicated that the participants preferred messages that were straightforward, enthusiastic, conversational, relatable, and authentic.

**Conclusions:**

This formative research has the potential to guide the design of messages for future digital health behavioral interventions targeting individuals from diverse racial and ethnic populations. Insights emphasize the importance of identifying key stimuli and tasks that require engagement, gathering multiple perspectives during message development, and using new approaches for collecting both quantitative and qualitative data while mitigating anonymity and confidentiality concerns.

## Introduction

### Background

Oral diseases constitute a significant public health issue and reflect social inequalities in health care [1]. Indeed, racial and ethnic minority populations are especially susceptible to poor oral health, largely due to barriers to accessing dental care and insurance coverage [2]. Although regular toothbrushing is considered a key self-care behavior necessary for sustaining good oral health, adherence to recommended brushing practices remains a challenge, particularly among individuals from low socioeconomic backgrounds [3]. Therefore, developing accessible and unobtrusive strategies to encourage regular oral self-care behaviors in at-risk groups could significantly lower the rates of dental disease and enhance health outcomes [4].

The ubiquity of digital technology, including mobile devices and wearable sensors, offers unique opportunities to expand access and enhance health outcomes, particularly among underserved populations [5]. With the ownership of smartphones approaching 85% within racial and ethnic minority populations [6], leveraging these cutting-edge devices can foster and encourage beneficial health behavior transformations in communities that have historically encountered health care inequities. Furthermore, digital tools facilitate the continuous tracking of a person’s condition and surroundings, thereby providing a means to offer timely and cost-effective digital prompts for health-related behaviors [7-9]. Despite the transformative promise of digital interventions, their true effectiveness is often undermined by lackluster engagement and the lack of concerted efforts toward their use [10-12]. Overcoming this significant hurdle is not just necessary but imperative to fully harness the power of digital health technologies and foster a more balanced and equitable health care environment.

In the realm of digital health interventions, the significance of formative work in crafting tailored messages cannot be overstated, particularly when addressing the unique needs and preferences of racial and ethnic minority groups. Well-designed mobile health tools that deliver culturally sensitive and personally relevant content can promote sustained engagement with the target behavior, thereby fostering positive health outcomes. By incorporating insights gleaned from formative research, digital interventions can have a greater impact than generic health promotion strategies by resonating with the target audience.

In this manuscript, we describe formative research undertaken to facilitate the creation of content for a digital oral health intervention specifically tailored to cater to an ethnically and racially diverse subpopulation. The messages, aimed at stimulating habitual oral hygiene practices in the morning and evening, will be delivered through a corresponding smartphone app, *Oralytics*. The app provides personalized messages and assists users in monitoring their brushing habits while presenting them with visual feedback on the duration and regularity of their brushing sessions.

Drawing on existing perspectives on engagement in digital interventions [9], message content was designed to foster engagement in 3 critical aspects: the targeted behavior (morning and evening brushing), the mobile app, and the messages themselves. We begin by discussing the strategies used to promote engagement in each dimension. Next, we describe the videoconference-based participatory co-design approach used to develop and refine the messages; this approach involves dental experts, individuals from the general population, and individuals from the target population—dental patients, predominantly from low-income racial and ethnic minority groups. Finally, we delve into a methodological approach used to address ethical considerations associated with the use of digital platforms (ie, Zoom [Zoom Video Communications]) for conducting internet-based discussion groups during the COVID-19 pandemic. Insights gleaned from this formative research have the potential to inform and shape the design of messages for future digital health behavior interventions.

### Creating Template Messages

A recent framework for engagement in digital interventions [9] encourages careful consideration of the question, “engagement with what?” when developing strategies for promoting engagement. Specifically, digital interventions often use multiple digital stimuli and tasks as a vehicle for engaging individuals with other tasks, nondigital or digital. As different strategies may be needed to increase engagement in different stimuli or tasks, an important first step is to clearly specify the key aspects that require engagement. Here, the focus is on engagement with the target behavior, the mobile app, and the mobile-delivered messages.

Our primary focus was on promoting engagement with the target behavior—oral self-care practices. Clinical guidelines typically endorse toothbrushing twice daily, in the morning and evening, as the most effective preventive routine [13]. Thus, the goal was to design messages that serve as a subtle cue for brushing by making oral health salient in the morning and evening. Second, given that the *Oralytics* app provides feedback on brushing practices (eg, time spent brushing and how well each quadrant of the teeth was brushed) essential for improving oral self-care, our goal was to design the messages in a way that also promotes engagement with the *Oralytics* app. Finally, our goal was to ensure that the participants engaged with the messages themselves. Accordingly, the message content was designed to be efficient for the participant’s attention and cognitive resources while also being appealing and original.

In subsequent sections, we present each engagement strategy and detail the message characteristics used to operationalize each strategy into a digital prompt that promotes oral self-care practices. Table 1 provides examples of the messages.

**Table 1:** Sample engagement messages across 2 formative webinars.

Message type	Message	Follow-up if message clicked
Reciprocity	A gift awaits you! Redeem $0.5 from *Oralytics* towards your Prize Account!	N/A^a^
Reciprocity-by-proxy	You make the world a better place: Redeem $0.5 from *Oralytics* for your <<charity>> Account!	N/A
Question and answer	Do you ever have mouth sores?	Educational statements in *Oralytics*: If response=yes: Mouth sores can be really unpleasant. Brush twice a day, floss once a day, and use mouth wash to reduce the bacteria that can cause sores. If response=no: Great! Did you know that mouth sores can be caused by trauma, bacteria, viruses, or even ingredients in your food or toothpaste (for example, Sodium Lauryl Sulfate)?
Feedback	Want to know how well you’ve been brushing lately? Tap to view	Graphical feedback in *Oralytics*

^a^N/A: not applicable.

### Engagement With the Target Behavior

Focusing on prompting engagement with morning and evening oral health practices, we selected 3 theoretically grounded strategies that hold great promise for encouraging engagement in health behaviors and can be translated into a mobile health (mHealth) intervention setting: reciprocity, reciprocity-by-proxy, and curiosity.

#### Reciprocity

The concept of reciprocity suggests that providing an individual with a small, no-strings-attached reward has the potential to capitalize on an innate human tendency to return favors and acts of kindness [14-16]. This strategy can be translated into a digital intervention setting by delivering a prompt containing a small reward that is not contingent on the participant’s behavior [17]. This is expected to increase the likelihood that the participant will reciprocate by engaging with the behaviors encouraged by the digital intervention.

To operationalize this strategy, we developed messages that notify participants about a small US $0.5 gift that they can redeem for their “prize account.” These messages have the following structure: (1) be framed as a US $0.50 gift from *Oralytics* to ensure participants understand that *Oralytics* is the source of the gift and the target for reciprocation and (2) include the word “Redeem” to signal the need for individuals to click on the message for the US $0.50 to be added to their prize account. At the end of the intervention, individuals will earn a gift card for the amount of money accrued in their prize account.

#### Reciprocity-by-Proxy

The related concept of reciprocity-by-proxy [18] centers on the notion of providing an unsolicited, no-strings-attached reward or benefit to a third party that an individual values (ie, considers important and desirable) to amplify the person’s sense of indebtedness and obligation to reciprocate. The strategy can be adapted for use in digital interventions by prompting participants with information about an unconditional donation that was made on their behalf to a charity they care about. This is expected to increase the likelihood that participants will reciprocate by engaging in the behaviors encouraged by the intervention.

To operationalize this strategy, we developed messages that notify participants about a small, no-strings-attached US $0.50 gift that they can redeem for their “charity account.” These messages have the following characteristics: (1) they are framed as a US $0.50 gift from *Oralytics* to ensure participants understand that *Oralytics* is the source of the gift and the target for reciprocation; (2) the charity that each participant selected (during an onboarding session) is explicitly mentioned to highlight that the donation was made to the person’s valued charity, and (3) the word “Redeem” is included so that individuals know that they must click directly on the message for the US $0.50 to be added to their charity account. At the end of the study, *Oralytics* will donate to the charity of the participant’s choice, in an amount equal to the funds accumulated in their charity account.

#### Curiosity

The concept of curiosity is defined as “a desire to acquire new knowledge and new sensory experience that motivates exploratory behavior” [19]. This desire can be leveraged in digital intervention settings by delivering prompts that contain questions, generate inquiry, and encourage inquisitive thinking related to the target behavior [20-22]. Such prompts are expected to increase engagement in the target behavior.

To operationalize curiosity, we developed 2 types of messages. The first type was designed to provide information to improve individuals’ oral health knowledge. The goal was to create messages that contain a question relevant to the participant’s current oral health. Answering the question with either “yes” or “no” will trigger the *Oralytics* app to display relevant, tailored educational information and advice. These question and answer (Q&A) messages have the following characteristics: (1) they contain an oral health–related question that can be answered with either yes or no, and (2) the answer triggers a follow-up statement with educational information or advice tailored to the participant’s answer. The second type of curiosity message was designed to encourage participants to access graphically displayed feedback in the *Oralytics* app (see the Engagement With the Mobile App section below for further description).

### Engagement With the Mobile App

To increase engagement with the *Oralytics* app, we designed a second type of curiosity message that encouraged participants to access feedback about their brushing behaviors within the app. The goal was to create messages that are nonevaluative, nondirective, and generally supportive of an individual’s autonomy [23-26]. Specifically, these feedback messages were designed to adhere to the following characteristics: (1) encourage the participant to seek out and access feedback on their brushing behaviors and (2) communicate autonomy support by emphasizing that the individual has a choice of whether to view their feedback. This autonomy support is accomplished by using suggestive (eg, consider viewing your brushing statistics) rather than directive (eg, you must check your brushing statistics now) framing.

Beyond the design of the curiosity messages, all messages were designed to facilitate participant interactions with the *Oralytics* app. Specifically, the reciprocity and reciprocity-by-proxy messages were designed such that participants were guided to click on the message to redeem the gift in the app. The Q&A curiosity messages were designed such that clicking either yes or no in response to the question would direct the participant to the appropriate answer within the app. Finally, the feedback curiosity messages were designed to direct the participant to the location of their brushing feedback in the app (ie, the participant can view the feedback in the app by clicking on the message).

### Engagement With the Messages

To promote engagement with the messages, their content was designed in a way that is resource efficient (in terms of the participant’s attention and cognitive processing), appealing, and novel.

#### Resource Efficiency

Individuals must navigate a large amount of information in their daily lives, which can often tax attentional and cognitive resources. Given this high volume of information, individuals are more likely to read and click on brief messages than messages that require a great deal of attention and concentration to read and understand [27]. Thus, all messages were designed to be brief in length (ie, no longer than 140 characters) and to use relatively simple language to improve comprehension and reduce cognitive burden.

#### Message Appeal

Messages that are framed in positive and encouraging ways are likely to enhance positive mood and approach motivations [28], where approach motivations reflect a drive or an “impulse to go toward positive stimuli” [29]. Thus, a message that is framed in a positive and generally upbeat manner is likely to encourage people to actively engage with (eg, fully read or click on) the message. Furthermore, by increasing engagement with the messages, a positive tone may also enhance the salience of and promote more positive attitudes toward brushing. Such positive attitudes may further encourage engagement with brushing and the *Oralytics* app. Given these numerous plausible benefits, all messages within the 4 categories were designed to be positive, encouraging, and enthusiastic in tone.

#### Message Novelty

A collection of messages were generated for each message category to increase message novelty and reduce habituation over time. Specifically, for each category, we generated messages that adhered to the characteristics described earlier but still varied in other respects. For example, the feedback messages were framed in one of three ways: (1) as a question (to communicate autonomy support), (2) as a statement highlighting the benefits of viewing feedback (to communicate that the feedback is useful and relevant to the participant), or (3) as a general statement (to provide a simple reminder that feedback is available in the app). In the following section, we describe the process of further developing and refining these messages.

## Methods

### Message Development and Refinement

After generating an initial collection of messages for each category (see examples in Table 1), we used a videoconference-based participatory co-design approach to further develop and refine the messages. This approach involved gathering and integrating insights from (1) dental experts via small groups of 4 to 6 dental residents and dental students; (2) individuals from the general population via Amazon Mechanical Turk (MTurk); and (3) dental patients drawn from community clinics who are representative of the target population via 2 facilitator-led videoconference sessions.

### Feedback From Dental Experts

Three generated message categories (reciprocity, reciprocity-by-proxy, and Q&A curiosity) were developed and refined based on feedback from small groups of 4 to 6 dental experts, including dental residents and dental students. Through meetings with experts, we obtained initial ratings of message liking and suggestions on how to improve the messages to make them more relevant and appealing to dental patients. The meetings provided invaluable feedback for the initial development and refinement of the messages. However, these meetings did not focus on the second category of curiosity messages (ie, feedback messages), as they were relatively simple and straightforward and did not require dental expertise. To ensure that the messages were understandable and appealing to the general population, it was critical to obtain additional feedback from nonexperts who were more similar to individuals who would be targets of future mHealth digital oral self-care interventions.

### Feedback From MTurk Employees

The use of MTurk workers provided an affordable and efficient way to collect initial message ratings that were balanced by key demographic characteristics (eg, gender, racial, or ethnic minority status). The MTurk phase also allowed for the generation of additional messages by people in the general population, which provided key insights beyond those of the authors and dental experts.

All 4 message categories were pretested with MTurk workers via the CloudResearch Platform. The CloudResearch Platform helps to exclude scam workers and ensure higher quality worker responses. Two web-based pretest sessions were conducted with MTurk workers located in the United States. In the initial session, 100 MTurk workers (n=50, 50% female; n=33, 33% minority group) assessed the reciprocity, reciprocity-by-proxy, and Q&A curiosity message types. In a second session, 50 MTurk workers (n=25, 50% female; n=17, 34% minority group) assessed the second type of curiosity messages (ie, feedback curiosity messages). MTurk workers in both sessions rated each message using the following criteria: (1) how much they liked or disliked the message, (2) how likely they thought another person would be to tap on the message, (3) whether the length of the message was acceptable, and (4) the message grammar.

To assist MTurk workers with assessing the probability of an individual tapping on a given message, we initiated each session with a context-setting scenario. The scenario featured an individual named “Alex,” whose gender was not specified. Alex had difficulties maintaining a consistent toothbrushing routine and was participating in an intervention program aimed at improving this habit. Messages would be sent once or twice daily to motivate Alex and other participants. These messages encouraged them to engage with “Oralytics,” a dedicated oral health smartphone app. This scenario was framed to provide the context for message delivery so that workers could evaluate them even if they do not personally struggle with oral self-care. Furthermore, we asked workers to evaluate messages in terms of their length and grammar, in addition to liking, because these aspects play an important role in message processing and appeal [30-32]. Although we could have incorporated other criteria, such as evaluating the novelty of the educational content for the worker or the potential of the message framing to incite preferable actions, our objective was to keep the message-rating task concise. This was done to prevent overburdening the MTurk workers, thereby reducing potential fatigue and facilitating more meaningful ratings. If any worker indicated that the message required grammatical cleanup, they were prompted to provide suggestions for correcting the grammar. The MTurk workers were also given the option of generating additional messages. On the basis of these insights, messages were identified for inclusion in the facilitator-mediated group webinars 1 and 2. The message inclusion criteria and webinar methods are described in detail in subsequent sections.

### Feedback From Dental Patients

Focus groups are typically defined as a group interview involving explicit interaction among group members that is intended to help explore people’s experiences, attitudes, and knowledge of some topic of interest [33]. Although traditional focus groups allow group members to communicate with each other, they may also involve ethical challenges given privacy concerns that arise when participants directly interact and share their opinions with each other [34]. The COVID-19 pandemic has increased such concerns, as many focus groups conducted during the time of quarantine moved to videoconference formats, where the participants’ names and locations (eg, the inside of their homes) are often visible [35-37]. Dos Santos Marques et al [37] took steps to mitigate some of these concerns by providing participants with anonymous names in Zoom focus group sessions. However, the participants were still asked to turn their cameras on during the session. Although participants can use web-based background features to control what their camera reveals [38], they may still have limited control over the behavior of other people in their physical environment (eg, family, roommates, and a partner), which may lead to a privacy breach. These considerations are particularly important when conducting web-based research with economically and socially vulnerable populations because of the fear of social stigma and discrimination [39,40]. To mitigate these concerns, we adopted a facilitator-mediated approach to conduct 2 videoconference sessions with dental patients. This approach, which we describe in detail in the Procedure section, enabled participants to provide feedback and generate suggestions during the sessions, while shielding their identities.

The participants were recruited from a pool of patients obtaining care at a large community dental clinic. A large proportion of the dental community clinic patients are from low-income racial and ethnic minority groups and are a key target demographic for the digital oral health intervention. Table 2 summarizes the sociodemographic characteristics of the participants. Although 15 participants were scheduled for the second session, only 7 attended.

**Table 2:** Participant characteristics for videoconference session 1 and 2.

Category and characteristics		Webinar session 1 (n=13)	Webinar session 2 (n=7)
Session date		March 2022	July 2022
Age (y), median (range)		30-49 (18-50+)	30-49 (30-50+)
**Sex, n (%)**			
	Female	5 (38)	4 (57)
	Male	8 (62)	3 (43)
**Race and ethnicity, n (%)**			
	Asian	2 (15)	—^a^
	Black or African American	3 (23)	7 (100)
	White (non-Hispanic)	1 (8)	—
	Hispanic	7 (54)	—

^a^No data were collected from individuals who identify with these racial and ethnic groups.

### Ethical Considerations

The procedures involving the participants were performed in accordance with the 1964 Helsinki Declaration and its later amendments. The research protocol was reviewed and approved by the institutional review board (IRB) at UCLA (IRB#20-000106).

Informed consent was obtained from all webinar participants. The participants were adequately informed about the study, and all their questions were answered before their involvement. The consent procedure included information about the purpose, procedures, potential risks and benefits of participation, and participants’ rights, including their right to withdraw at any time without any penalties. The original informed consent documents explicitly allowed for the potential use of the collected data for future research without requiring additional consent, in line with IRB guidelines.

To mitigate privacy concerns and comply with the IRB policies on protecting the identity of participants during web-based research sessions, we developed and refined messages through 2 facilitator-mediated formative sessions using a videoconferencing platform (Zoom). Each participant was only able to see and hear the facilitator with whom they interacted through Zoom polls and a chat window anonymous to other participants. Participants were not able to interact with each other directly and thus could not see any identifying information from fellow participants. This strategy effectively shielded the participants’ identities during the sessions.

Participants in both webinars were compensated for their time and efforts with a US $25 Amazon gift card upon completion of the session. This form of compensation was guided by the need to respect participants’ time and efforts while avoiding undue inducement.

All study data were anonymized and deidentified to protect the privacy and confidentiality of the participants. When necessary, additional protective measures were applied, such as data encryption and restricted access.

### Message Inclusion

The first webinar session focused on the reciprocity, reciprocity-by-proxy, and Q&A curiosity message types. It included 20 messages (5, 5, and 10, for each message type, respectively) that were rated below average during the first MTurk session and were thus revised based on MTurk worker feedback. Focusing on the revised messages minimized participant fatigue and kept the session to 1 hour in length. The follow-up webinar session (session 2) focused only on the feedback curiosity messages. As these messages were not evaluated by dental experts, we generated 52 feedback curiosity messages for the second MTurk session with the intention of dropping the lowest rated 22 messages from further consideration. The remaining 30 messages were selected and revised based on MTurk worker feedback and included in webinar session 2. The session duration was similar to session 1.

### Procedure

Each session used a combination of methods to collect quantitative and qualitative feedback about the messages. First, the webinar facilitator explained that the purpose of the session was to refine messages in preparation for an intervention that sought to encourage people to use (engage with) an oral health smartphone app. Messages were presented to participants one at a time, and participants were asked to rate each message on the following scale: 1=do not like it; 2=like it somewhat; and 3=like it. Messages were rated using the Zoom webinar polling feature. For any message that received a rating of “1=Do not like it,” participants were given the opportunity to send the facilitator (through the Zoom webinar chat feature) suggestions for improving the message. Participants could also provide reactions or further feedback in response to key suggestions read aloud to the group by the facilitator. Participants volunteered their suggestions and were not cold-called or otherwise required to provide feedback. Therefore, not all participants responded with suggestions for every message. Screenshots of all polls were taken for later data entry, and all participant chat messages were saved.

## Results

### Quantitative

The mean ratings of reciprocity (mean 2.65, SD 0.52), reciprocity-by-proxy (mean 2.58, SD 0.53), Q&A curiosity (mean 2.45, SD 0.69), and feedback curiosity (mean 2.77, SD 0.48) message types indicated that all were rated highly, with a high overall message rating collapsing across types (mean 2.63, SD 0.58; Multimedia Appendix 1).

The means of the 4 message types ranged between mean 2.08 (SD 0.76; corresponding closely to “Like it somewhat”) and mean 3.00 (SD 0.00; corresponding to “Like it”). On the basis of feedback given by participants during the 2 webinars, a threshold of 2.50 was identified, whereby any messages that received a mean rating of mean 2.50 to 3.00 (60% reciprocity; 80% reciprocity-by-proxy; 40% Q&A curiosity; 90% feedback curiosity; 76% overall) were not further modified. This threshold was identified because it fell between the “Like it somewhat” and “Like it” ratings and >75% of messages, collapsing across the 4 categories, fell at or above this rating. Messages that received an average rating of <2.50 (40% reciprocity, 20% reciprocity-by-proxy, 60% Q&A curiosity, 10% feedback curiosity, and 24% overall) were revised using the webinar participant suggestions as a guide. One exception was a feedback curiosity message that despite receiving a high average rating (mean 2.71) also had a relatively high SD of 0.76 and received critical participant feedback during the webinar. Thus, this message was also revised for clarity.

### Qualitative

Participant feedback varied according to each specific message type. For the reciprocity and reciprocity-by-proxy messages, quantitative ratings showed that participants generally did not dislike these message types. This meant that there were fewer suggestions for improvement as participants were only prompted for feedback when they rated a message as “1=Do not like it” during the webinar polling. In the first facilitator-led webinar, only 1 reciprocity message (and none of the reciprocity-by-proxy messages) received a participant rating of “1=Do not like it.” One participant suggested making the app’s intentions clearer in the message. This could be interpreted as a need for clarity that the app is intended for oral self-care. However, as reciprocity messages focused on the no-strings-attached reward provided by the app, this feedback was used to emphasize that the reward was a gift from *Oralytics* without any conditions rather than a reward for a specific behavior. Reciprocity and reciprocity-by-proxy messages with ratings below the identified mean 2.50 threshold were also modified to be less complex, even if they did not receive a rating of “1=Do not like it” during the session. For example, the lowest rated (mean 2.31) reciprocity-by-proxy message: “Think about the impact you can have, <<Name>>. Redeem a $0.5 gift from *Oralytics* to your <<Charity>> Account!” did not receive any ratings of “1=Do not like it” and therefore participants were not prompted to provide feedback on this message. However, given the low average rating, this message was revisited and appraised by the researchers as being relatively complex because it asked the individual to think about their possible impact. To simplify, the message was updated as follows: “You are making an impact, <<Name>>. Redeem a $0.5 gift from *Oralytics* to your <<Charity>> Account!”

The participants provided more suggestions for the curiosity messages. We also obtained feedback on the answers (ie, educational statements) provided in *Oralytics* after participants responded yes or no to a Q&A curiosity message. Participant feedback was greater for the Q&A curiosity messages than for the feedback curiosity messages. Table 3 provides examples of qualitative participant suggestions for 3 Q&A messages and 2 feedback curiosity messages.

Across both webinar sessions, participant suggestions for modifying the message language fell into the following categories: (1) more straightforward (less confusing or complex); (2) more conversational (less formal); (3) enthusiastic (eg, with an exclamation point); (4) avoid mention of specific actions that may not be relatable to everyone (eg, how to seek a prescription to treat sleep apnea); (5) avoid phrases that could be interpreted as disingenuous (eg, “sorry to hear that”); and (6) for some message types (ie, feedback curiosity) specific to the domain of oral health.

**Table 3:** Example message updates based on participant suggestions.

Type and message		Answers^a^	Examples of participant suggestions	Suggestions interpretation	Revised message	Revised answers^a,b^
**Question and answer curiosity**						
	“Do you have any tooth pain?”	If response=yes: “We are sorry to hear this. If it persists for more than a day, make an appointment with your dentist as soon as you can. In the meantime, try to rinse with salt water or apply a cold compress.” If response=no: “This is good news! Often if you wait until your teeth hurt to see a dentist it’s more difficult to treat a cavity or gum disease.”	“never like to hear sorry to hear this.” “if no starting with ‘that’s great’ sounds weird” “You can say ‘Ouch! Tooth pain is never fun’”	Language that seems odd or disingenuous, like “sorry to hear this” should be avoided.	N/A^c^	If response=yes: “*Tooth pain is never fun*^b^. If it persists for more than a day, make an appointment with your dentist as soon as you can. In the meantime, try to rinse with salt water or apply a cold compress.” If response=no: “*Glad to hear it!* Often if you wait until your teeth hurt to see a dentist it’s more difficult to treat a cavity or gum disease.”
	“Do you sleep well at night?”	If response=yes: “That’s great! Sleeping well at night is important to your health, including your dental health. Make sure to keep your stress levels low and get enough sleep each night.” If response=no: “Not sleeping well at night may be a sign of sleep apnea. Sleep apnea can cause breathing from one’s mouth at night, which can lead to more plaque retention on your teeth and make you susceptible to cavities. Your dentist can prescribe sleeping devices for sleep apnea.”	“Better to say ‘Do you get enough sleep?’ like it’s a number rather than a feeling” “yes it should be worded as how much sleep over how well” “If no shouldn’t say you can get the prescription from the dentist. It almost sounds like you’re suggesting that person to get the prescription. like Q2 you should just say contact your dentist.”	Frame questions about sleep (or similar behaviors) in terms of quantity over quality. Avoid recommending courses of action that may not be generally relatable.	Do you *get enough sleep* at night?	If response=yes: “That’s great! *Getting enough sleep* at night is important to your health, including your dental health. Make sure to keep your stress levels low *to* get enough sleep each night.” If response=no: “Not *getting enough sleep* at night may be a sign of sleep apnea. Sleep apnea can cause breathing from one’s mouth at night, which can lead to more plaque retention on your teeth and make you susceptible to cavities. *Check with your doctor or dentist if you continue to have problems sleeping*.”
	“Have you had something sugar sweetened to eat or drink today?”	If response=yes: “Occasional added sugar is ok when part of a balanced diet. Brush tonight to reduce the bacteria that feeds on sugar in your mouth.” If response=no: “Making healthy food choices is important. A balanced diet that limits added sugar will help you achieve a healthy smile!”	“Maybe change the question to ‘Have you had something with added sugars to eat or drink today?’” “I like ‘added sugar’ better” “either sounds fine.” “really liked both answers” “Yeah, the suggestion is a more common way of saying it”	Questions should be worded in a common or conversational style (how people speak).	Have you had something *with added sugar* to eat or drink today?	N/A
**Feedback curiosity**						
	“Brush up on your *Oralytics* feedback”	Graphical feedback provided in the app	“Here how you did on your oralytics”	The message should be straightforward, without using phrases such as “brush up” that not everyone may understand.	*Click here to view your Oralytics* feedback.	N/A
	“Learn how you brush to improve your health. Click to view.”	Graphical feedback provided in the app	[Add] “Improve oral health”	Clarify that the feedback is specifically about oral health.	Learn how you brush to improve your *oral* health. Click to view.	N/A

^a^These are educational statements sent by *Oralytics* tailored to the participant’s response to a Q&A message.

^b^Updates are italicized in the “Revised message” and “Revised answers” columns.

^c^N/A: not applicable.

## Discussion

### Overview of Findings

The formative studies described here sought to develop and refine messages for a digital oral health intervention. These messages were designed to promote engagement in (1) morning and evening oral health practices, by highlighting reciprocity, reciprocity-by-proxy, and curiosity; (2) the mobile app, by creating messages that encourage the participant to access and view feedback on their brushing behaviors in the *Oralytics* app; and (3) the messages themselves, by creating content that is resource efficient, appealing, and novel.

To develop the messages, we first identified the specific (digital and nondigital) stimuli and tasks that require engagement to achieve the ultimate goal of the digital oral health intervention. Next, we identified theoretically grounded strategies that have the potential to enhance engagement in each identified stimulus or task. Third, guided by the selected engagement strategies, we created message templates, namely protocols that outline the specific characteristics of each category of messages. These templates were used to generate an initial set of messages for each category. To promote greater message engagement, their content was designed to be resource efficient (low burden in terms of attention and cognitive processing), appealing, and novel. Finally, to further develop and refine the collection of messages, we used a web-based participatory co-design approach involving several methods, including gathering and integrating feedback from small groups of dental experts, using MTurk to gather insights from the general population, and further evaluation and refinement by dental patients through facilitator-mediated group webinar sessions. We found this multistage approach to provide highly valuable feedback that highlighted aspects of the messages that needed improvement. This feedback was then used to facilitate the development of messages that have the potential to be highly engaging.

Participants recruited for the group webinar sessions were primarily from underserved racial and ethnic minority groups, which reflects the general sociodemographic makeup of the Los Angeles metropolitan area and is generally representative of the target population for the planned intervention study. Over the course of these group webinar sessions, we found that all 4 categories of messages were highly rated, with the lowest average rating for any given message corresponding near the scale label of “like it somewhat.” Participants offered more suggestions for enhancing the Q&A curiosity messages compared with other types of messages. This could be attributed to several factors. First, Q&A curiosity messages are relatively complex, as they require individuals to respond to a personal oral health–related question. Second, these messages often elicit longer answers. Third, the evaluation process included 2 components: the message itself and the corresponding answers. Finally, this type of message generally received lower numerical ratings (as shown in Multimedia Appendix 1), which led the facilitator to seek additional feedback.

Overall, the qualitative feedback suggests that participants preferred messages that were more straightforward, enthusiastic, conversational, relatable, authentic, and, for some message types (ie, feedback curiosity), specific to oral health. Through these group webinar sessions, we identified which messages needed final refinement and made relatively minor modifications to this subset of messages using suggested feedback from participants.

The facilitator-mediated group webinars used in this research combined both qualitative and quantitative methods that allowed participants to provide message ratings and feedback without access to each other’s names, faces, or other immediately identifying information. We found these procedures useful in developing and refining digital intervention messages with racial and ethnic minority groups. This approach holds promise for increasing population reach and providing enhanced anonymity and confidentiality to underserved groups.

### Limitations

Although not knowing the names and identities of other participants in the sessions may enhance anonymity and confidentiality, it is currently unclear to what extent participants in the study perceived this to be the case. For instance, it is still possible for participants to infer general sociodemographic information from the type and content of peer feedback provided, even if read by the facilitator. Furthermore, although participants did not have access to peer names and other identifying information, the facilitators, who were university-affiliated White women, viewed participants’ names during the sessions. Future research should thus seek to determine whether this facilitator-mediated group webinar approach indeed leads participants from racial and ethnic minority groups to perceive greater anonymity and confidentiality and in turn to feel more comfortable providing honest feedback relative to traditional focus groups. Future research should also determine whether this kind of facilitator-mediated group webinar has an advantage relative to other qualitative methods, including individual interviews. For instance, it could be that participants in facilitator-mediated group webinars feel less pressure to provide socially desirable responses than participants in either individual interviews or focus group settings, which could improve the accuracy and generalizability of their responses.

An important limitation relates to the approach used to elicit feedback from webinar participants. Specifically, the facilitator asked for suggestions only if a message was rated “1=Do not like it” by the participant. More feedback could have been obtained by asking participants for comments regardless of the quantitative ratings they provided. However, doing so would have substantially increased the length of the webinar and facilitated a discussion that was less focused on the messages that were most critical to refine. Future research should develop new strategies for eliciting rich feedback in a manner that is also focused and resource efficient.

Another limitation of this research is the uneven sample size across the 2 facilitator-mediated group webinar sessions, with the first session including nearly double the sample size of the second session. This discrepancy was due to scheduled participants not attending the second webinar session, resulting in the sample being comprised entirely of African American participants. It would have been ideal to have a larger sample size that represented a greater diversity of racial and ethnic minority identities. However, there is no reason to suspect that the appeal of feedback messages would be substantially different among African Americans compared with other underserved minority groups. Future research should seek to identify the best way to recruit a broad and diverse range of racial and ethnic minority participants. Future research should also involve the target population in the very initial process of identifying and translating engagement strategies so that their perspectives, skills, knowledge, and expertise can contribute to the entire process of message development [41,42].

### Next Steps

The messages described here will be used in a microrandomized trial [43-45] study with 70 dental patients to empirically optimize the delivery of just-in-time support [21,46] for oral self-care. The goal of this microrandomized trial is to investigate whether delivering a message, what type of message, and under what conditions delivering a message increases engagement in ideal oral self-care. The results will build the empirical foundation necessary to develop an optimized digital intervention that delivers the right type of message at the right time to promote oral self-care in the home setting.

### Conclusions

This formative work has the potential to guide the development of messages for digital interventions that target individuals from racial and ethnic minority groups. It emphasizes the importance of identifying the key stimuli and tasks that require engagement, involving the perspectives of multiple people (eg, participants, dental experts, and researchers) in the process of message development, and using new approaches for collecting both quantitative and qualitative data while plausibly enhancing participant anonymity and confidentiality. Future research should continue to elucidate the best approaches for refining mHealth messaging designed to support racial and ethnic minority populations.

## Appendix

Multimedia Appendix 1 Mean ratings of the 4 message types. Q&A: Question and Answer.

## Abbreviations

IRB: institutional review board
MTurk: Mechanical Turk
Q&A: question and answer

## Data Availability

Deidentified data sets generated and analyzed during this research are available in the Open Science Framework repository [47].
