# Supplementary figures and images for: Developing Message Strategies to Engage Racial and Ethnic Minority Groups in Digital Oral Self-Care Interventions: Participatory Co-Design Approach
Source: JMIR Form Res. 2023 Dec 11;7:e49179. doi: 10.2196/49179 (PMC10750234; doi:10.2196/49179)

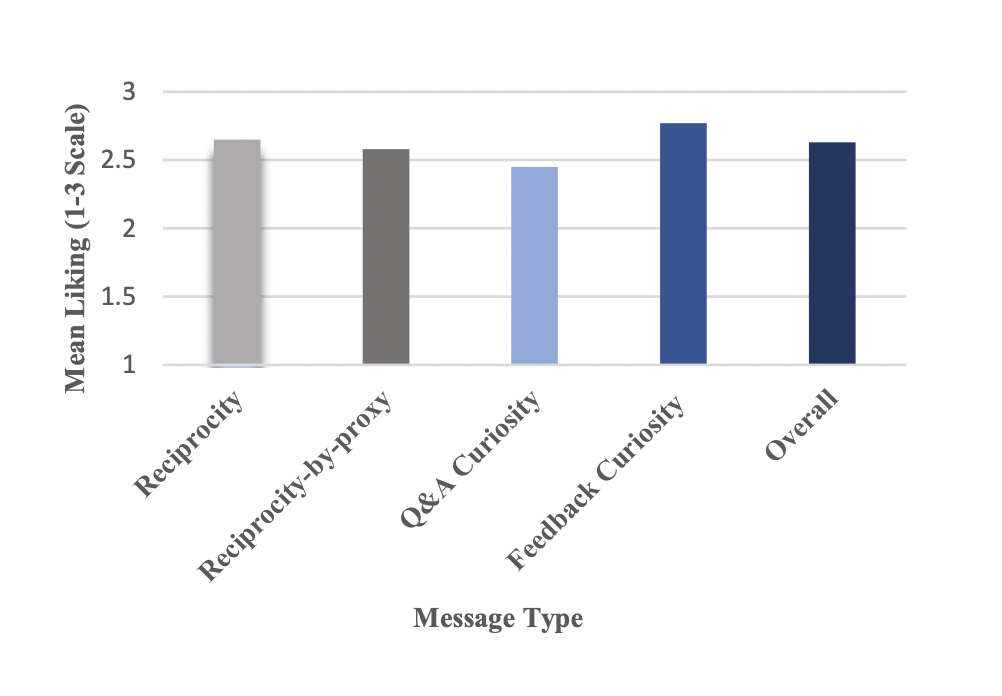

Supplement: Multimedia Appendix 1 [file formative_v7i1e49179_app1.png]
